# Hydroxyapatite Nanorods Based Drug Delivery Systems for Bumetanide and Meloxicam, Poorly Water Soluble Active Principles

**DOI:** 10.3390/nano14010113

**Published:** 2024-01-02

**Authors:** Valeria Friuli, Lauretta Maggi, Giovanna Bruni, Francesca Caso, Marcella Bini

**Affiliations:** 1 Department of Drug Sciences, University of Pavia, Viale Taramelli 12, 27100 Pavia, Italy; lauretta.maggi@unipv.it; 2 Department of Chemistry, University of Pavia, Viale Taramelli 16, 27100 Pavia, Italy; giovanna.bruni@unipv.it (G.B.); francesca.caso01@universitadipavia.it (F.C.); marcella.bini@unipv.it (M.B.); 3 CSGI—Department of Chemistry, University of Pavia, Viale Taramelli 16, 27100 Pavia, Italy; 4 National Reference Centre for Electrochemical Energy Storage (GISEL)—INSTM, Via G. Giusti 9, 50121 Firenze, Italy

**Keywords:** hydroxyapatite, meloxicam, bumetanide, drug delivery, inorganic–organic hybrids, in vitro dissolution studies, solubility

## Abstract

Poorly water-soluble drugs represent a challenge for the pharmaceutical industry because it is necessary to find properly tuned and efficient systems for their release. In this framework, organic–inorganic hybrid systems could represent a promising strategy. A largely diffused inorganic host is hydroxyapatite (HAP, Ca_10_(PO_4_)_6_(OH)_2_), which is easily synthesized with different external forms and can adsorb different kinds of molecules, thereby allowing rapid drug release. Hybrid nanocomposites of HAP nanorods, obtained through hydrothermal synthesis, were prepared with two model pharmaceutical molecules characterized by low and pH-dependent solubility: meloxicam, a non-steroidal anti-inflammatory drug, and bumetanide, a diuretic drug. Both hybrids were physically and chemically characterized through the combined use of X-ray powder diffraction, scanning electron microscopy with energy-dispersive spectroscopy, differential scanning calorimetry, and infrared spectroscopy measurements. Then, their dissolution profiles and hydrophilicity (contact angles) in different media as well as their solubility were determined and compared to the pure drugs. This hybrid system seems particularly suitable as a drug carrier for bumetanide, as it shows higher drug loading and good dissolution profiles, while is less suitable for meloxicam, an acid molecule.

## 1. Introduction

Inorganic–organic hybrids, constituted by an inorganic matrix and a drug, represent, in the last few years, one of the most promising systems of drug delivery for the pharmaceutical industry [1,2]. This is particularly true for poorly water-soluble drugs, which are a challenge for pharmaceutical scientists, and unfortunately a large number of newly discovered drugs are slightly water-soluble. After the oral administration of a drug in a solid form (tablets, capsules, or suspensions) and before adsorption, it is necessary for it to be released and then dissolved in the gastrointestinal fluids [3,4]. The dissolution rate is the limiting step of the bioavailability of a large number of poorly water-soluble drugs. This parameter is in turn related to the surface area available for dissolution: in the case of a dissolution rate slower than the absorption rate, the dissolution step clearly becomes the rate-limiting step in the absorption process [5]. To improve the speed and completeness of the adsorption of problematic drugs, many strategies have been proposed, such as particle size reduction and the preparation of solid dispersions or salts, micelles, or liposomes [6,7,8]. However, inorganic–organic hybrids stand out compared to the other proposed systems due to their many intriguing features. In fact, inorganic matrices may be used as carriers for controlled and/or targeted drug release as well as to improve the drugs’ long-term stability and storage by protecting them from unsafe environments. However, the drug-loaded amount is the most critical parameter for acceptable industrial applications, and it should be improved by properly tuning the inorganic systems. The most interesting inorganic matrices are represented by hydroxyapatites. Hydroxyapatite (*HAP*, Ca_10_(PO_4_)_6_(OH)_2_) is the main constituent of biological tissues, such as bones and teeth [9]. It is biocompatible, biodegradable, osteoconductive, and bioactive and can bond to living tissues and both positive and negative molecules through simple absorption [10,11]. *HAP* was proposed as a carrier for targeted drug delivery due to its low solubility in physiological conditions. It can also minimize the toxicity to other organs, reduce the drug amount in the blood, and avoid the repeated administration of drugs. It has been reported in many studies that *HAP* is neither toxic to organs nor cytotoxic [12,13]. Hydroxyapatite is easily synthesized with different external forms, which could have an influence on drug loading and subsequent release. The most diffused are the spherical nanoparticles, but nanorods, flower-like, plate-like, and dumbbell-like nanoparticles are also reported [14]. The different surface areas and the possible porosities obtained during synthesis are fundamental parameters for the efficacy of drug release systems based on *HAP*, with the adsorption phenomenon being at the basis of hybrid formation. An electrostatic interaction can take place, as well as hydrogen bond interactions involving the OH^−^ groups of *HAP* [15]. 

Poorly water-soluble drugs can pertain to different pharmacological classes. For example, bumetanide, a diuretic, and meloxicam, an anti-inflammatory drug, both pertain to Biopharmaceutical Classification System (BCS) class II, i.e., drugs with low solubility and high permeability.

Bumetanide (*Bum*) (brand names: Bumex^®^ and Burinex^®^, 3-(butylamino)-4-phenoxy-5-sulfamoylbenzoic acid, C_17_H_20_N_2_O_5_S) is a loop diuretic that can be applied to treat congestive heart failure, hepatic and renal diseases, and mild hypertension [16,17]. It works by decreasing the reabsorption of sodium by the kidneys; the target site of *Bum* is the Henle loop ascending limb and, thanks to its diuretic and natriuretic effect, can induce quick, pronounced diuresis [18,19]. This drug, being 40 times more effective than the better-known furosemide (common name: Lasix^®^), is considered an effective option for individuals for whom furosemide does not work appropriately. *Bum* is practically insoluble in water, with an increase in solubility with a decreasing pH; it is instead more soluble in many organic solvents, such as ethanol, dimethyl-formamide (DMF), and dimethyl-sulfoxide (DMSO) [20].

Meloxicam (*Mlx*, 4-Hydroxy-2-methyl-N-(5-methyl-2-thiazolyl)-2H-1,2-benzothiazine-3-carboxamide-1,1-dioxide, C_14_H_13_N_3_O_4_S_2_) is a non-steroidal anti-inflammatory drug (NSAID). It pertains to the oxicam class and shows analgesic and antipyretic properties. It can be used to treat arthritis and osteoarthrosis for a limited time and can treat rheumatoid arthritis for longer times [21]. *Mlx* has five polymorphic forms, as reported in the literature [22], but form I is commercialized [23]. Meloxicam is insoluble in water, slightly soluble in methanol and ethanol, and highly soluble in DMF and DMSO [24]. 

The low solubility that characterizes these drugs represents a big problem during the formulation process and can explain the differences in oral bioavailability. 

In this paper, the results of the synthesis of hydroxyapatite-based hybrids of both bumetanide and meloxicam are discussed. These systems, to our knowledge, are being proposed for the first time for both these drugs. A complete physical–chemical characterization was first performed for the drugs and hydroxyapatite and then for the hybrids to demonstrate their formation by means of X-ray powder diffraction (XRPD), Fourier transform infrared spectroscopy (FT-IR), differential scanning calorimetry (DSC), and scanning electron microscopy with energy-dispersive spectroscopy (SEM-EDS). Pharmaceutical measurements, in particular the dissolution rates in different biorelevant media simulating gastrointestinal environments and the solubility and contact angle values, demonstrated the suitability of the hybrids to act as drug delivery systems.

## 2. Materials and Methods

**Scheme 1:**
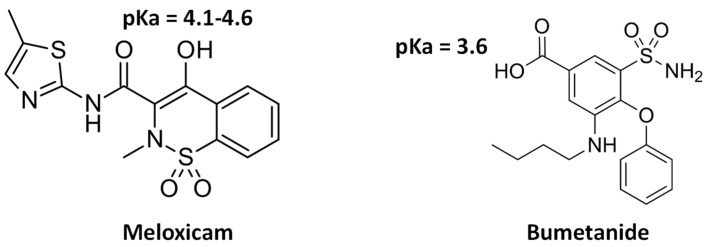
Molecular structures of meloxicam and bumetanide active principles.

Meloxicam (*Mlx*) and bumetanide (*Bum*) (Scheme 1) were kindly donated by Olon (Casaletto Lodigiano, LO, Italy) and Fidia Farmaceutici S.p.A. (Abano Terme PD, Italy), respectively. 

A suitable medium to solubilize the drugs, which are both very poorly water-soluble, was necessary for the hybrids’ preparation. The medium that was found after many trials was a mixture of ethanol/water (3:1 *v*/*v* ratio) for both the drugs. 

### 2.1. Hydroxyapatite Synthesis

Hydroxyapatite was synthesized via hydrothermal synthesis. The required amounts of (NH_4_)_2_HPO_4_ (0.2 g) and Cethyl-ammonium bromide (CTAB) (0.7 g) were dissolved in 20 mL of distilled water with magnetic stirring at about 400 rpm for several minutes. Some drops of NaOH (2M) were added to reach a pH ranging between 9.5–11. Then, the solution was sonicated for about 30 min. In another beaker, CaCl_2_ 4H_2_O (0.46 g) was dissolved in 20 mL of water. After complete dissolution, the solution of calcium was added drop by drop to the solution of phosphorus, and the final solution was heated to 60 °C and maintained at this temperature for about 4 h under vigorous agitation. The solution was then transferred in a Teflon beaker, hermetically sealed in a stainless-steel container, and treated in oven at 180 °C for 18 h. Finally, the powder was collected via centrifugation, washed 2 times with distilled water, then treated overnight at 60 °C in an oven. 

This sample was named *HAP-NRD*.

### 2.2. Hybrid Preparation

The preparation was the same for both drugs. For the preparation of the hybrids, solutions of the drugs were prepared: first, 200 mg of meloxicam or bumetanide was added to 20 mL of a mixture of ethanol/water (3:1 *v*/*v*) and magnetically stirred until complete drug dissolution. The addition of one or two drops of base was useful to complete the dissolution. Then, 300 mg of *HAP-NRD* was added to the drug solution, sonicated for about 5 min, and stirred at room temperature for 24 h. The dispersion was then centrifuged at 6000 rpm for 5 min, and the powder was dried in an oven at 60 °C overnight. 

The two hybrids were named *HAP-NRD-Mlx* and *HAP-NRD-Bum*.

### 2.3. Physical–Chemical Characterizations

A Bruker D5005 diffractometer (Bruker BioSpin, Fällanden, Switzerland) equipped with Cu Kα radiation, a graphite monochromator, and a scintillation detector was used to perform X-ray powder diffraction (XRPD) measurements. The patterns were collected in air with the following conditions: a step size of 0.03° and a counting time of 2 s per step in different angular ranges (5–40° (*Mlx and Bum*) and 10–70° (*HAP-NRD* and hybrids)) using a silicon sample holder.

The Fourier transformed infrared (FT-IR) spectra were obtained using a Nicolet FT-IR iS20 spectrometer (Nicolet, Madison, WI, USA) with the ATR (Attenuated Total Reflectance) sampling accessory (Smart iTR with diamond plate). The spectra were obtained by co-adding 32 scans in the 4000–400 cm^−1^ range at a 4 cm^−1^ resolution.

Differential scanning calorimetry (DSC) measurements were performed on a DSC Q2000 apparatus interfaced with a TA 5000 data station (TA Instruments, New Castle, DE, USA). Ultrapure (99.999%) indium (m.p. = 156.6 °C; Δ*H* = 28.54 J g^−1^) was used as a standard to calibrate the instrument. The calorimetric measurements were performed up to 280 °C (heating rate: 5 K min^−1^) under nitrogen flow (45 mL·min^−1^) on samples weighing about 3–5 mg in open standard aluminum pans. 

A Zeiss Evo MA10 (Carl Zeiss, Oberkochen, Germany) microscope coupled with an energy-dispersive spectroscopy (EDS) detector for microanalysis (X-max 50 mm, Oxford Instruments, Wiesbaden, Germany), working at an acceleration voltage of the electron beam of 20 kV, was used to collect scanning electron microscopy (SEM) images and microanalysis spectra. The samples for the SEM analysis were sputtered with gold, while the samples for EDS were used as they were.

### 2.4. Pharmaceutical Measurements

#### 2.4.1. Drug Loading

The drug loading of the *HAP-NRD-Mlx* and *HAP-NRD-Bum* hybrids was determined by placing exactly weighed quantities of the hybrid compounds in flasks containing a known volume of a fluid in which the drug is very soluble and “in sink conditions” (phosphate buffer (pH = 7.5) for *Mlx* and deionized water for *Bum*) and leaving them under continuous stirring. After time intervals, the UV-Vis absorbance (Lambda 25, Perkin-Elmer, Monza, Italy) was measured on a filtered portion of the fluid, and this was repeated until the readings were constant over time.

Calibration curves were determined in the different fluids that were considered, with a correlation coefficient of 0.9999. The UV spectrum of the inorganic carrier was previously recorded to verify that it did not exhibit any absorbance at the same wavelength as the active substances.

#### 2.4.2. Dissolution Test

The dissolution tests were performed in some biorelevant fluids chosen to simulate the in vivo condition of oral administration. 

The dissolution tests were performed on the actives and hybrid compounds in a powder previously sieved through a 230-mesh grid (63 μm) (Endecotts, London, UK) using a USP apparatus 2 and a paddle (Erweka DT-D6, Erweka GmbH, Dusseldorf, Germany) at 37.0 ± 0.5 °C (three replicates). 

*Mlx* and *HAP-NRD-Mlx* were tested in 900 ml of phosphate buffer (pH 7.5) according to an official monograph reported in the US Pharmacopoeia [25], 0.1 N HCl (pH 1.0) (simulating the gastric environment in fasted conditions), phosphate buffer (pH 4.5) (simulating the gastric environment in fed conditions), and deionized water (simulating consumption with a glass of water) at 75 rpm. All the samples contained a 7.5 mg dose of *Mlx.*

*Bum* and *HAP-NRD-Bum,* containing a 2 mg dose of the active substance, were tested in 900 mL of deionized water according to an official monograph reported in the US Pharmacopoeia [26], the same volume of 0.1 N HCl (pH 1.0), and phosphate buffer (pH 4.5) at 50 rpm.

The *Mlx* and *Bum* concentrations were measured using a UV-Vis spectrophotometer (Lambda 25, Perkin-Elmer, Monza, Italy) on filtered portions of the dissolution fluid at 362 and 274 nm, respectively. The data were processed using suitable software (Winlab V6 software, Perkin-Elmer, Monza, Italy). All the dissolution media were prepared according to the “Reagent and buffer solutions section” of the USP [27].

#### 2.4.3. Solubility

The solubility of *Mlx, Bum,* and the hybrids was measured in deionized water at 21 °C using the shake-flask method. At predetermined time intervals, an aliquot of the supernatant was filtered through 0.22 μm Millipore filters. The drug concentration was determined via UV detection, and the test was repeated until equilibrium was reached. The results were the average of three replicates ± the standard deviation (SD).

#### 2.4.4. Contact Angle

Images of drops in contact with samples of *Mlx, Bum,* and the hybrid compounds, *HAP-NRD*-*Mlx* and *HAP-NRD-Bum*, in powder, were acquired at various times (from t = 0 up to 300 s) using a Contact Angle Meter DMe-211Plus (NTG Nuova Tecnogalenica, Cernusco, Italy). Drops (10 µL) of different fluids (0.1 N HCl (pH 1.0), phosphate buffer at pH 4.5 and pH 7.5, and deionized water) were dropped from a needle, and images of the drops on the surfaces of the samples were acquired. The data were processed using suitable software provided by the equipment. Three replicates were performed for each sample.

## 3. Results and Discussion

The combined use of different physical–chemical techniques, applied first to the drugs and hydroxyapatite alone, then to the hybrids, was the winning strategy to verify the formation of the hybrids. In the following sections, the XRPD, FT-IR, DSC, and SEM-EDS results will be discussed, as well as the pharmaceutical results, which were particularly useful to test the efficacy of the hybrids for the improvement of the dissolution rate and solubility with respect to the pure drugs.

### 3.1. Physical–Chemical Characterization 

**Figure 1:**
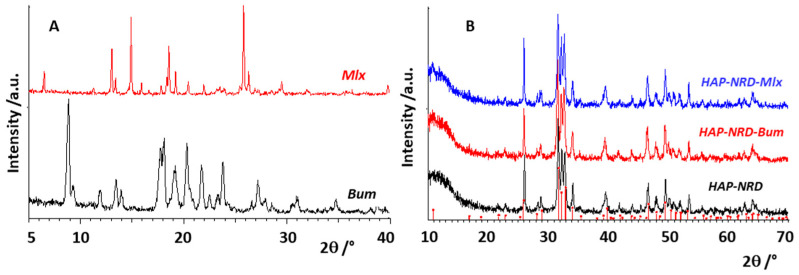
X-ray diffraction patterns of (**A**) *Mlx* and *Bum* drugs and (**B**) *HAP-NRD*, *HAP-NRD-Bum*, and *HAP-NRD-Mlx*. In (**B**), the red vertical bars represent the expected peak positions of *HAP* (Card No. 74-0565).

The polymorphic phase, the purity, and the morphological features were first determined for the drugs and hydroxyapatite. The XRPD patterns of the drugs and *HAP-NRD* are shown in Figure 1A,B, respectively. 

Meloxicam and bumetanide are crystalline drugs whose patterns (Figure 1A) agree well with polymorphic form I and the triclinic form reported in the literature, respectively [23,28]. Hydroxyapatite has the expected pattern (Figure 1B), which is well explained by the bars of the peak positions of the hexagonal polymorph (*P6_3_/m* space group) (Card No. 74-0565). No peaks due to secondary phases are present, suggesting a high-purity inorganic carrier. 

The peak positions and intensities of the patterns of the hybrids are similar and resemble those of pure *HAP-NRD* (Figure 1B). No peaks due to the drugs are evident. This observation suggests that, after the adsorption on the *HAP* surface, both the drugs turned out to be amorphous or that their amounts were under the minimum detection limit. This last hypothesis, however, seems less reliable due to the high sensitivity of the XRPD technique towards crystalline phases.

**Figure 2:**
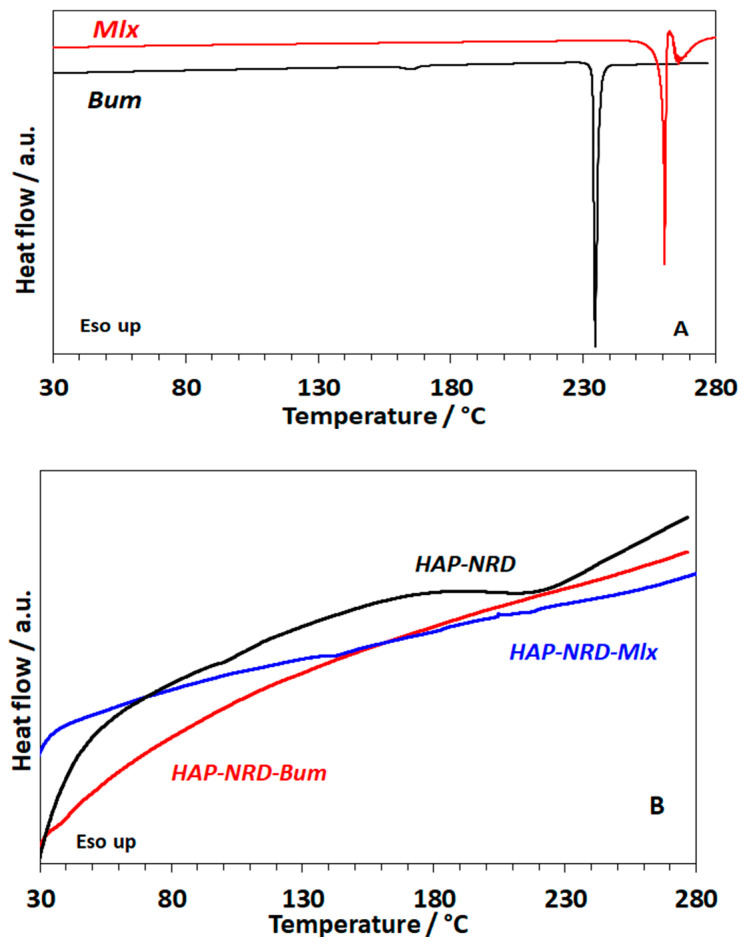
DSC curves of (**A**) *Mlx* and *Bum* drugs and (**B**) *HAP-NRD* and hybrids.

The DSC curves of both drugs are reported in Figure 2A. *Bum*, at about 160 °C, shows a transition from polymorph II to the polymorph I crystal structure, then an endothermic peak at about 239 °C due to the melting of form I [20]. *Mlx* only shows an endothermic peak of melting at about 265 °C, followed by rapid decomposition [29]. *HAP-NRD* (Figure 2B), a highly stable ceramic material, does not present any thermal events in the analyzed temperature range, apart from a broad endothermic peak possibly due to the release of adsorbed water or an initial dehydroxylation [30].

The DSC curves of the hybrids are also reported in Figure 2B. They can be useful to investigate the possible presence of the drugs and their thermal stability in the hybrids by exploring the presence of melting peaks, the only thermal event possibly presents in the curve due to the high stability of the inorganic component *HAP*, as previously demonstrated. The recorded curves (Figure 2B) do not present any thermal event; the melting of both drugs is no longer visible (Figure 2A). Therefore, we can suppose that the adsorbed drugs are amorphous, in agreement with the XRPD results. 

**Figure 3:**
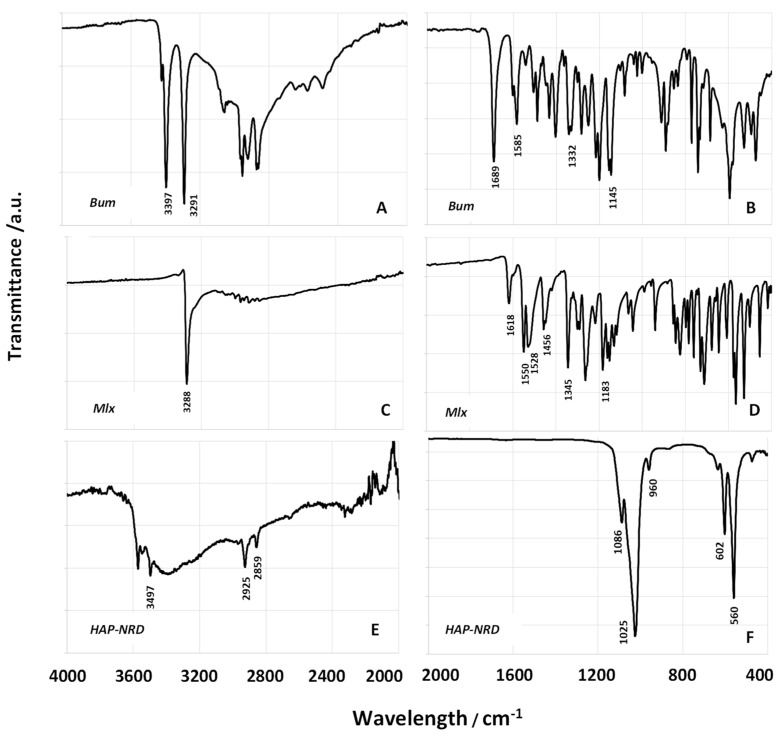
FT-IR spectra of (**A**–**D**) *Mlx* and *Bum* drugs and (**E**,**F**) *HAP-NRD*.

The FT-IR spectra confirm the findings of the DSC and XRPD techniques (Figure 3). 

*Bum* (Figure 3A,B) has main vibrational peaks at 3397 cm^−1^ and 3291 cm^−1^ (bands of amine I sulphonamide and NH asymmetric and symmetric stretching), 1689 cm^−1^ (carboxylic band), 1585 cm^−1^ (N-H vibration), and 1345 and 1145 cm^−1^ (asymmetric and symmetric stretching of SO_2_) [20]. *Mlx* (Figure 3C,D) has main absorptions at 3288 cm^−1^ (NH stretching), 1618 cm^−1^ (stretching of amide C=O), 1550–1528–1456 cm^−1^ (stretching of aromatic ring), and 1345 cm^−1^ and 1183 cm^−1^ (asymmetric and symmetric stretching of SO_2_) [29]. The *HAP-NRD* spectrum (Figure 3E,F) shows the typical phosphate vibrational bands: the bending of the O-P-O group is responsible for the peaks at 560 cm^−1^ and 602 cm^−1^, the band at 960 cm^−1^ is due to the stretching of the PO_4_^3−^ group, and asymmetric P-O stretching produces the bands at around 1000 cm^−1^. The peaks at 3497 cm^−1^ and about 630 cm^−1^ (very low) are due to O–H stretching and bending, respectively [31]. In addition, the bands at around 2925 and 2859 cm^−1^ could be due to a residual of CTAB, the complexant used during the synthesis [32].

**Figure 4:**
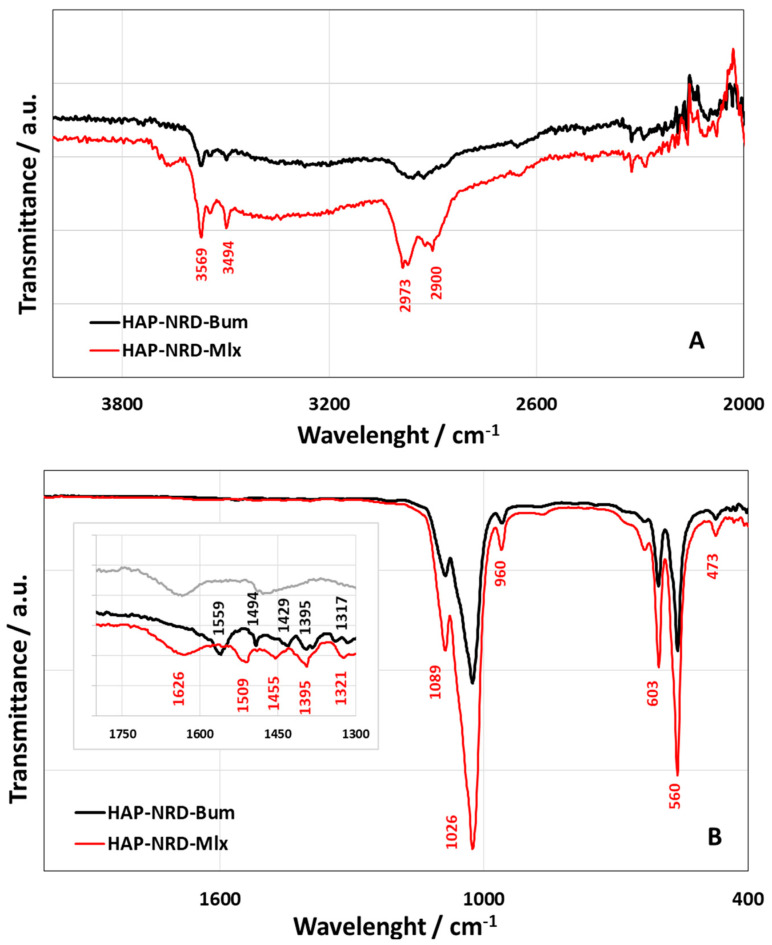
FT-IR spectra of *HAP-NRD-Bum* and *HAP-NRD-Mlx* in the spectral ranges 4000–2000 cm^−1^ (**A**) and 2000–400 cm^−1^ (**B**). In (**B**), the inset shows a magnification of the 1800–1300 cm^−1^ range, with the spectrum of *HAP-NRD* (in grey) also reported for comparison.

The FT-IR spectra of both hybrids are compared in Figure 4A,B. 

At first glance, the spectra resemble that of pure *HAP-NRD* (Figure 3E,F): the main vibrational peaks are in fact those of the phosphate group of hydroxyapatite (at about 1000 and 600 cm^−1^). However, the magnification of the 1800–1300 cm^−1^ region, where the bands typical of drugs can be expected, shows interesting hints (Figure 4B, inset). Apart from the peak at about 1626 cm^−1^, which is also present in the *HAP-NRD* spectrum, the other small peaks are clearly pertinent to drugs. For *HAP-NRD-Bum*, the peaks at about 1559 cm^−1^ and 1317 cm^−1^ correspond to those at 1585 and 1345 cm^−1^ for the pure *Bum*. The shifts to lower wavenumbers are compatible with an interaction between the drug and *HAP*, in particular a hydrogen bond between the OH groups of *HAP* and the COO- of *Bum* [15]. In fact, the peak at about 3497 cm^−1^ in the *HAP-NRD* spectrum, due to OH stretching (Figure 3E), is slightly shifted and lowered in the hybrid, supporting its involvement in a hydrogen bond with the drug. The band at 1689 cm^−1^ of the carboxylic group is no longer visible, justifying the conversion of the carboxylic acid in the corresponding carboxylate, with typical peaks at about 1540 and 1370 cm^−1^, present as shoulders of other peaks. The same is true for the *HAP-NRD-Mlx* hybrid’s spectrum: the peaks at 1509–1455–1395 cm^−1^ are attributable to the aromatic ring of *Mlx*, while the peak at 1321 cm^−1^ can correspond to that at 1345 cm^−1^. Also, in this case, the shifts of the vibrational bands are compatible with an electrostatic interaction between *HAP-NRD* and *Mlx*, as suggested for other drugs [15]. In the spectra of *HAP-NRD* and the hybrids (Figure 3F and Figure 4B), the absorption bands of CO_3_^2−^ at 1480 cm^−1^, associated with the symmetric stretching mode, and at about 860 cm^−1^, due to the out-of-plane bending mode (this last one is more evident), are also present [33]. 

Therefore, from the FT-IR spectra we could demonstrate the presence of the drugs, even if probably in low amounts. This technique is in fact susceptible to the presence of peculiar functional groups but is not dependent on the crystalline forms of the compounds.

**Figure 5:**
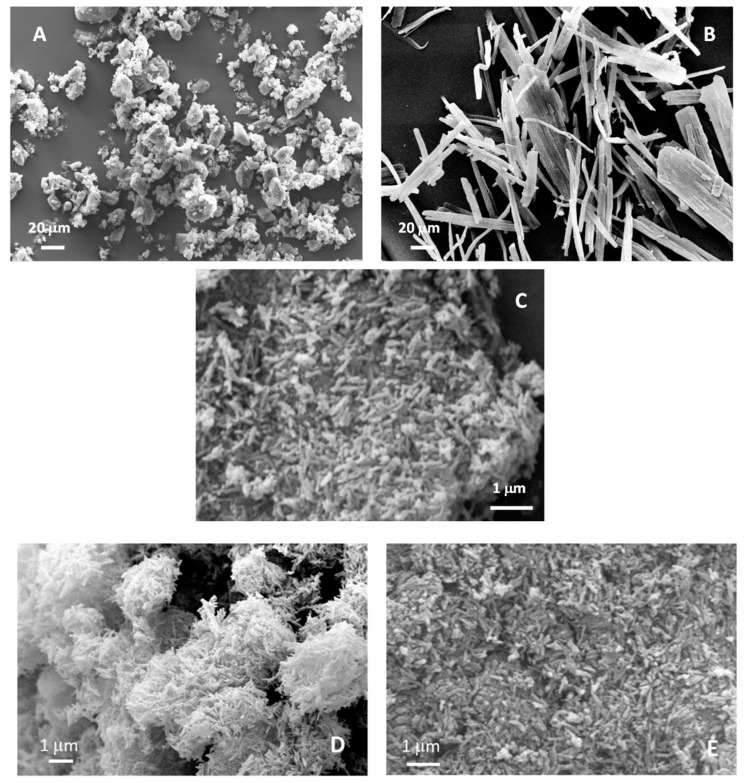
SEM micrographs of (**A**) *Mlx*, (**B**) *Bum*, (**C**) *HAP-NRD*, (**D**) *HAP-NRD-Bum*, and (**E**) *HAP-NRD-Mlx* samples.

The morphology of the samples was studied using SEM (Figure 5). 

*Mlx* (Figure 5A) shows particles of variable sizes and a prevalent spherical form measuring between 1 and 20 µm. *Bum* (Figure 5B) has particles with a classic needle-like form, with lengths of tens of microns and a flat section. *HAP-NRD* (Figure 5C) shows large aggregates of small nanorods well below 1 μm in length, whose dimensions strictly depend on the molar ratio of CTAB/Ca^2+^ used during the synthesis [34]. It was reported that the lengths of the nanorods decrease by increasing the CTAB amount. In our case, the ratio was about 1.32:1 (see Section 2.1).

The hybrids (Figure 5D,E) are constituted by aggregates of smaller particles with elongated forms, i.e., nanorods. The morphology resembles that of the pure *HAP-NRD* (Figure 5C), and no variation was introduced after the drugs’ adsorption. There is no evidence of particles of drugs, which are particularly characteristic, at least for *Bum*. The EDS analysis allowed us to determine Ca/P ratios of 1.62 and 1.51 for *HAP-NRD-Bum* and *HAP-NRD-Mlx*, respectively, in good agreement with the stoichiometric values (1.67). 

### 3.2. Pharmaceutical Characterization

**Figure 6:**
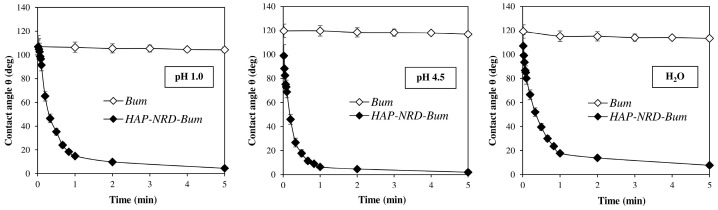
Contact angle, θ, of *HAP-NRD-Bum* compared to *Bum* alone in the different biorelevant fluids considered.

The drug loading of *HAP-NRD-Bum* was not high: 3.3 ± 0.2 %. Its water solubility was 40.2 ± 1.3 mg/L, which was almost twice that of *Bum* (23.3 ± 2.3 mg/L). The *HAP-NRD-Bum* hybrid’s wettability was significantly improved compared to the pure drug *Bum* (Figure S1 and Figure 6). The contact angle between the fluid and the powder decreased rapidly for *HAP-NRD-Bum* and reached zero in a few seconds, while, for the pure drug, it remained very high (110–120° θ) and did not decrease.

**Figure 7:**
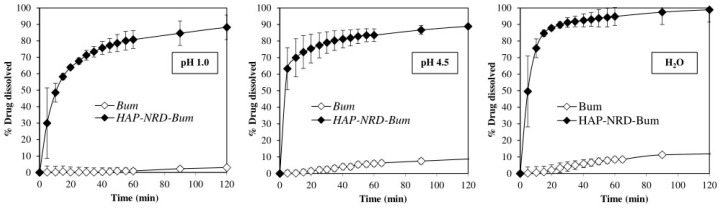
Dissolution profiles of *HAP-NRD-Bum* compared to *Bum* in the different biorelevant fluids considered. The dose is always 2 mg.

The dissolution profiles confirm a remarkable improvement in the rate at which the drug is made available in solution by *HAP-NRD-Bum* in all the biorelevant fluids considered, while *Bum* shows a very slow dissolution rate that quickly saturates under the same conditions (Figure 7). *Bum* is less soluble in an acidic solution, but the hybrid compound can release 90% of the dose at pH 1 (which simulates the fasted gastric environment) and pH 4.5 (which simulates the fed gastric condition) in about 2 h, while it can release 90% of the dose in water in about 30 min.

The solubility of both drugs depends on the pH and therefore on the fluid of the gastrointestinal environment that it will encounter after oral administration; for this reason, the dissolution tests were carried out both in water and "biorelevant" fluids prescribed by the pharmacopoeia to simulate these conditions. These fluids are generally buffers, so the pH was not modified by the presence of the sample, and indeed, at the end of each test, we verified that the pH remained constant. The behavior of the drugs during the dissolution tests was perfectly in line with the expectations: *Mlx* was very slightly and slowly soluble (pKa = 4.08) at a low pH and was much more soluble at a pH that was at least weakly basic (7.5). *Bum* (pKa1 = 3.6) was also very slightly and slowly soluble at acidic pHs. In any case, to obtain rapid absorption and therefore a therapeutic effect, both drugs should be absorbed in the first portion of the gastrointestinal tract and therefore in acidic conditions. Only in deionized water it was possible to verify a slight change in pH, which, however, was rather limited because the dose was very low for both drugs. In the case of *Mlx*, the pH of the deionized water decreased from 6.9 to 6.2, and in the case of *Bum*, the pH of the water decreased from 6.9 to 6.3.

For the *HAP-NRD-Mlx* hybrid, the drug loading was again not high at 2.7 ± 0.1%, which was slightly lower than that of *HAP-NRD-Bum*. But in this case, the *HAP-NRD-Mlx* solubility was indeed very high ,more than 115 mg/L, a level at which we had to stop the experiment because it became difficult to filter the suspension. The solubility of *Mlx*, measured in the same conditions, was found to be 7.9 ± 1.1 mg/L. 

Since *Mlx* is a hydrophobic compound, the drug wettability was low (120° θ) in all the fluids and remained constant over time (Figure S2), but in this case the wettability of *HAP-NRD-Mlx* was complete from the beginning of the test (0° θ).

**Figure 8:**
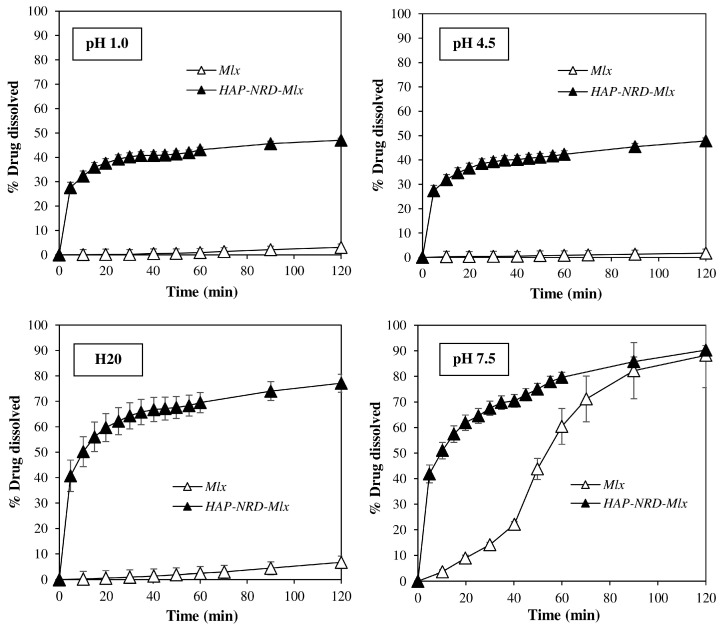
Dissolution profiles of *HAP-NRD-Mlx* compared to *Mlx* in the different biorelevant fluids considered. The dose is always 7.5 mg.

The solubility of *Mlx* is strongly pH-dependent and increases with an increasing pH: the dissolution was minimal at pH 1, less than 3% at pH 4.5, and less than 5% in water, while it increased significantly only in the phosphate buffer at pH 7.5 (simulating the intestinal environment) (Figure 8). This characteristic represents a big problem for the oral administration of this drug because it is not able to become effective until it reaches the intestinal tract, and this means a considerable delay of hours before pain relief. *HAP-NRD-Mlx*, on the other hand, showed notable increases in the dissolution rates of the drug in all the considered fluids. This means that it could release 50% of the dose in the fluid simulating the gastric environment in both the fasted (pH 1) and fed (pH 4.5) conditions and up to 75% of the dose in water (simulating intake with a glass of water). Finally, in the intestinal environment, the dissolution rate of *HAP-NRD-Mlx* was much faster compared to the pure drug. Although the new delivery system is not yet able to guarantee that the dose is released completely in three out of four media, it nevertheless represents a significant improvement compared to the pure drug. 

## 4. Conclusions

Two new hybrids based on hydroxyapatite nanorods loaded with two poorly water-soluble drugs, bumetanide and meloxicam, were successfully prepared for the first time. The presence of the drugs in an amorphous form and in small amounts in the hybrids was demonstrated via physical–chemical characterization. The pharmaceutical study showed large improvements in the dissolution rates, solubility, and wettability of the hybrids compared to the pure drugs, particularly for bumetanide and in acidic conditions, in which both drugs are less soluble. This improvement can be explained by several factors that act simultaneously: the amorphization of the drug, the increase in the active principle surface available for dissolution thanks to the fine dispersion in the nano-carrier, which significantly increases its wettability and avoids the agglomeration typical of hydrophobic products, and the attraction effect of the hydrophilic carrier towards water. The drug loading, the most critical parameter, could be improved via surface modifications of *HAP* (in particular the functionalization) or the addition of dopants in the crystal structure to increase the affinity of drugs towards the *HAP* surface. With a proper improvement in the adsorbed drug amount, we think that these organic–inorganic hybrids could be very attractive for the pharmaceutical market and could be applied to other BCS class II drugs. 

## Data Availability

The data are available upon request.

## Supplementary Materials

The following supporting information can be downloaded at https://www.mdpi.com/article/10.3390/nano14010113/s1, Figure S1: Contact angle images of *Bum* and *HAP-NRD-Bum* in different fluids at 0.5 min. Figure S2: Contact angle, θ, of *HAP-NRD-Mlx* compared to *Mlx* alone in the different biorelevant fluids.
