# Supplementary material for: Hydroxyapatite Nanorods Based Drug Delivery Systems for Bumetanide and Meloxicam, Poorly Water Soluble Active Principles
Source: Nanomaterials (Basel). 2024 Jan 2;14(1):113. doi: 10.3390/nano14010113 (PMC10780568; doi:10.3390/nano14010113)
Supplement: Supplementary file 1 [file nanomaterials-14-00113-s001.zip › nanomaterials-2745759-supplementary.pdf]

Hydroxyapatite nanorods based drug delivery systems for Bumetanide and Meloxicam, poorly water soluble active principles

Valeria Friuli, Lauretta Maggi, Giovanna Bruni, Francesca Caso, Marcella Bini

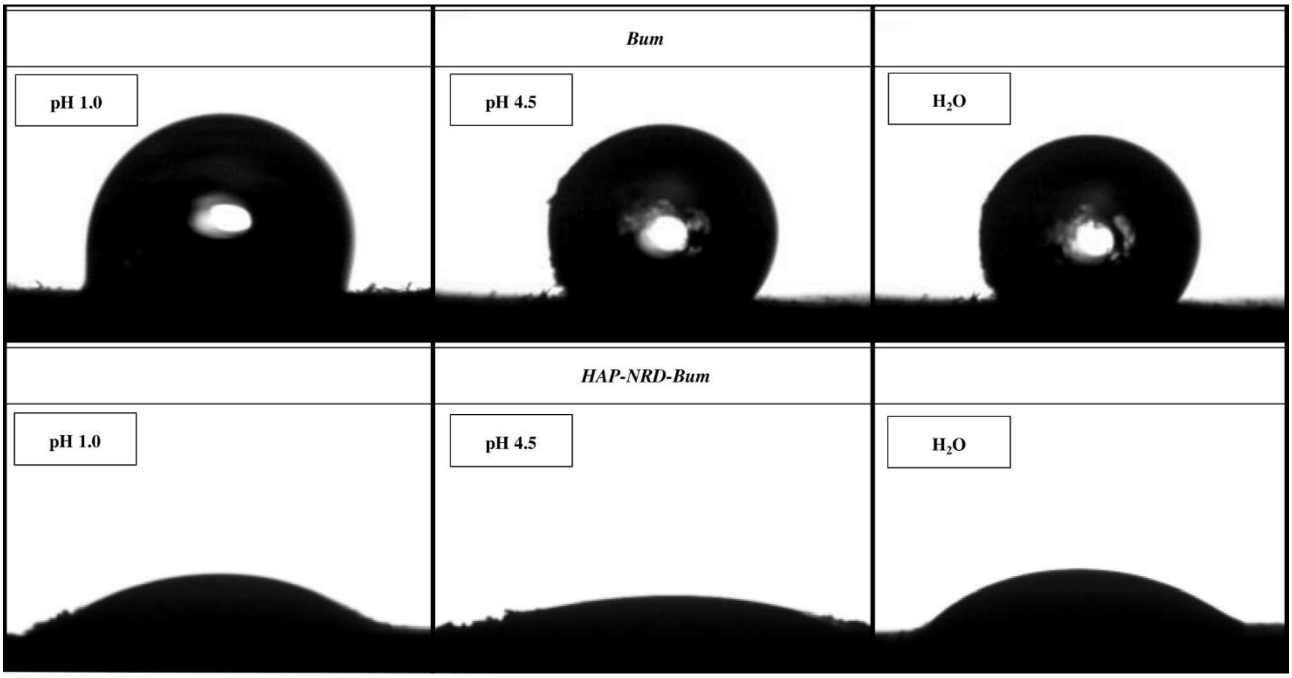

Figure S1 - Contact angle images of *Bum* and *HAP-NRD-Bum* in different fluids at 0.5 min.

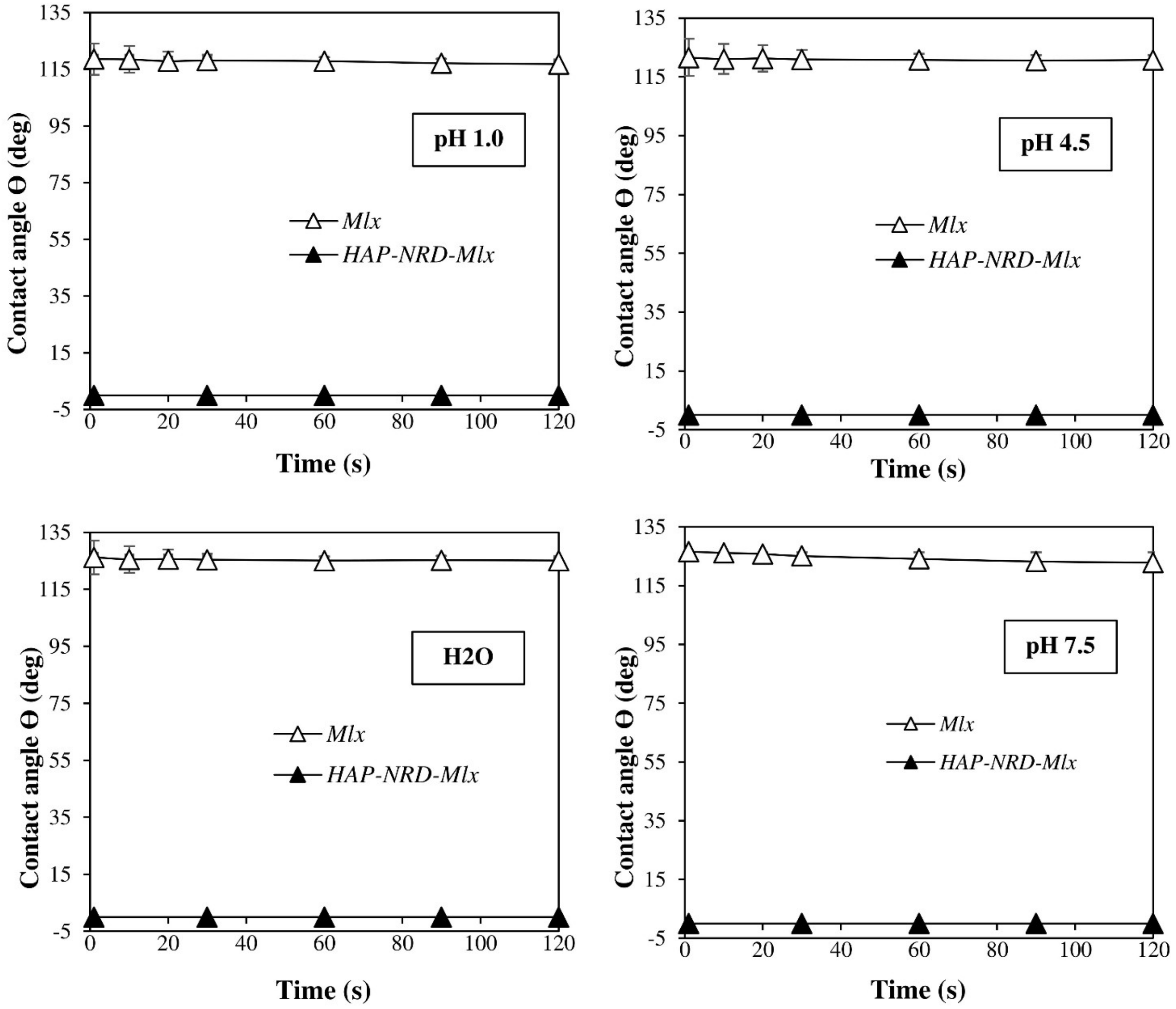

Figure S2 – Contact angle, θ, of HAP-NRD-Mlx compared to Mlx alone in the different biorelevant fluids.
